# The human milk bacteriome and mycobiome and their inter-kingdom interactions viewed across geography

**DOI:** 10.3389/fnut.2025.1610346

**Published:** 2025-07-07

**Authors:** Haipeng Sun, Brett Finlay, Meghan B. Azad, Christina A. Cuomo, Leah E. Cowen, Brittany Berdy, Jonathan Livny, Terrance Shea, Edna E. Aquino, Filipa Godoy-Vitorino, Melissa A. Woortman, Margot Shumaker, Claudio Alba, Juan M. Rodríguez, María G. Domínguez-Bello

**Affiliations:** ^1^Department of Biochemistry & Microbiology, Rutgers University, New Brunswick, NJ, United States; ^2^Department of Biochemistry & Molecular Biology, The University of British Columbia, Vancouver, BC, Canada; ^3^Humans and the Microbiome Program, Canadian Institute for Advanced Research, Toronto, ON, Canada; ^4^Manitoba Interdisciplinary Lactation Centre (MILC), Children’s Hospital Research of Manitoba and Department of Pediatrics and Child Health, University of Manitoba, Winnipeg, MB, Canada; ^5^Infectious Disease and Microbiome Program, Broad Institute of MIT and Harvard, Cambridge, MA, United States; ^6^Fungal Kingdom: Threats & Opportunities Program, Canadian Institute for Advanced Research, Toronto, ON, Canada; ^7^Department of Molecular Genetics, University of Toronto, Toronto, ON, Canada; ^8^Department of Microbiology, School of Medicine, University of Puerto Rico, San Juan, Puerto Rico; ^9^Department of Nutrition and Food Sciences, Complutense University of Madrid, Madrid, Spain; ^10^Pluridisciplinar Institute, Complutense University of Madrid, Madrid, Spain

**Keywords:** human milk, microbiome, bacteriome, mycobiome, global

## Abstract

**Background:**

The human milk microbiota is one of the biologically active components of human milk, and factors affecting it and the effect size are not well understood. Assessments of human milk microbiota have mainly been done in small cohorts and/or in single geographical locations, and most have been restricted to the bacteriome. Here we assessed the bacterial, archaeal and fungal composition of human milk and the potential inter-kingdom interactions in milk collected from women living in a wide spectrum of countries, environments, and socio-economical settings.

**Materials and methods:**

About 518 human milk samples were collected in 16 countries. After DNA extraction, bacterial and fungal metataxonomic analyses were performed via amplification and sequencing of the 16S rDNA and the ITS2 genes, respectively. In parallel, the presence of methanogenic archaea was determined by qPCR.

**Results:**

Bacterial analysis revealed significant Country variations in human milk microbiota diversity and taxa distribution. Core genera such as *Staphylococcus*, *Streptococcus*, and *Bifidobacterium* were universally prevalent, and their abundance varied geographically. Methanogenic sequences were found in the amplicon sequences, mostly of *Methanobrevibacter* (11.8% of samples), while qPCR only detected 0.7% (2 out of 268) methanogens. Fungi—mostly *Candida*—were detected in 7% of samples, with wide country variations. Co-abundance network analysis revealed mostly positive bacterial correlations and negative inter-kingdom interactions.

**Conclusion:**

This study shows substantial global variation in the human milk microbiome with bacterial-fungal interactions, highlighting the importance of global-scale studies to understand the human microbiome and its role in maternal and infant health.

## 1 Introduction

Human milk is the natural diet in early life, characterized by its unique and adaptable diversity of nutrients and bioactive components (1–3). In addition to proteins, carbohydrates and fats essential for infant nourishment, human milk contains hormones (4–8), immune factors (9–11), microbes (12–19) and microbial nourishment components such as human milk oligosaccharides (HMOs), which are indigestible for infants (20–22). Human milk represents one of nature’s most complex biological systems, and the mechanisms by which it influences infant development are currently being characterized (23–26).

Most research on human milk microbiomes has concentrated on bacteria and examined the impact of various factors including genetics, maternal age, diet, maternal BMI, mode of delivery, feeding practices, gestational age, and temporal changes (15–17, 27–30). Relatively few studies have explored non-bacterial microbes in human milk, such as archaea (31), fungi (32, 33), viruses (34–36), or assessed multi-kingdom microbial associations and their role in shaping microbial communities (37–40).

Limitations of studies so far include a focus primarily on bacterial communities, restricted sample sizes, and limited geographical diversity. Furthermore, variability in methods for sample collection, storage, and processing complicates determining the actual impact of any specific factor (41). Large-scale studies encompassing diverse geographic locations and socio-economic backgrounds, employing standardized methodologies, are necessary to characterize the variability of the human milk microbiome accurately. Such studies will help define distinct microbial community networks or “lactotypes” influenced by maternal, infant, and environmental factors (40, 42–45). This study aimed to contribute to a better knowledge of the bacterial, archaeal, and fungal composition of human milk and the potential inter-kingdom interactions in milk samples collected from women living in a wide spectrum of countries, including different environments and socio-economical settings.

## 2 Materials and methods

### 2.1 Subjects and sampling

Milk samples from 518 healthy mothers (one individual sample per mother) were obtained in 16 different countries, including cohorts from Equatorial Guinea (*n* = 33), Kenya (*n* = 30), Senegal (*n* = 60), South Sudan (*n* = 53), Tanzania (*n* = 4), Ecuador (*n* = 35), El Salvador (*n* = 6), Mexico (*n* = 18), Peru (*n* = 8), Puerto Rico (*n* = 5), mainland United States (*n* = 85), Austria (*n* = 38), Germany (*n* = 32), The Netherlands (*n* = 15), Norway (*n* = 20), and Spain (*n* = 76). The subject’s age, body weight, sampling time, and birth mode of the baby were listed in Table 1.

**Table 1:** The characteristics of the subjects by country.

Continent	Country	N	Maternal age (year)	Maternal weight (kg)	Sampling time post partum (day)	Birth mode
			N.not. NA, (Mean ± SD)	N.not. NA, (Mean ± SD)	N.not. NA, (Mean ± SD)	N.not. NA, (Vaginal, Cesarean)
Africa	Equatorial Guinea	33	33, (27.6 ± 5.2)	33, (64.3 ± 11.3)	33, (57 ± 20)	33, (28, 5)
	Kenya	30	30, (26.0 ± 5.2)	30, (60.7 ± 10.4)	30, (75 ± 23)	30, (23, 7)
	Senegal	60	60, (27.3 ± 6.1)	60, (60.3 ± 10.3)	60, (65 ± 16)	60, (60, 0)
	South Sudan	53	53, (23.6 ± 4.6)	53, (53.9 ± 7.1)	53, (60 ± 16)	48, (48, 0)
	Tanzania	4	4, (29.3 ± 7.2)	3, (42.7 ± 1.5)	0, (NA)	0, (NA)
America	Ecuador	35	35, (27.7 ± 6.1)	35, (65.9 ± 12.7)	35, (62 ± 17)	35, (20, 15)
	El Salvador	6	6, (28.0 ± 8.4)	0, (NA)	0, (NA)	6, (4, 2)
	Mexico	18	18, (29.5 ± 5.1)	18, (76.9 ± 11.3)	18, (62 ± 23)	18, (11, 7)
	Peru	8	8, (24.6 ± 5.0)	8, (61.8 ± 10.9)	8, (55 ± 16)	8, (2, 6)
	Puerto Rico	5	0, (NA)	5, (62.3 ± 13.5)	0, (NA)	5, (4, 1)
	United States	85	85, (32.8 ± 4.2)	24, (73.0 ± 14.9)	55, (57 ± 47)	85, (51, 34)
Europe	Austria	38	38, (32.6 ± 5.1)	0, (NA)	0, (NA)	38, (28, 10)
	Germany	32	32, (28.9 ± 4.8)	32, (74.9 ± 13.4)	32, (68 ± 17)	32, (25, 7)
	Netherlands	15	15, (29.0 ± 3.8)	0, (NA)	0, (NA)	15, (11, 4)
	Norway	20	20, (30.9 ± 5.2)	20, (73.0 ± 12.1)	20, (51 ± 18)	20, (16, 4)
	Spain	76	76, (34.5 ± 3.7)	40, (65.2 ± 9.5)	40, (70 ± 25)	76, (68, 8)
Total		518	513, (29.6 ± 6.0)	361, (64.4 ± 13.0)	384, (63 ± 26)	509, (399, 110)

The study procedures related to the samples obtained from Equatorial Guinea, Kenya, Senegal, South Sudan, Ecuador, El Salvador, Mexico, Peru, Austria, Germany, The Netherlands, Norway, and Spain were approved by the overarching Institutional Review Board of the European Commission in the frame of the EU project “Variations in biochemical and microbiological milk composition among highly diverse human populations and their impact on infant gut ecosystem” (call FP-7-PEOPLE-2013-IEF) (protocol #624773, approved on 14 February 2014) and at each study location, and consent was obtained from each participating woman. Human milk samples from Puerto Rico were approved by the University of Puerto Rico, Medical Sciences Campus (IRB ProB2310120, approved on 24 March 2021). The United States samples were approved by the Rutgers University-New Brunswick Health Sciences Institutional Review Board (Protocol #Pro2018002781, approved on 17 September 2021; Protocol #Pro2020002169, approved on 9 June 2021), and consent was obtained from each participating woman. The samples from Tanzania are from a previous study approved by the IRB reference number 164-12-21052012 (46).

Collection of human milk was performed by the mothers as described by McGuire et al. (47); briefly, the aureola skin was wiped with antiseptic wipes containing chlorhexidine digluconate (bactiseptic wipes, Vesismin Health, Barcelona, Spain) using gloved hands, and milk was manually expressed into disposable sterile containers. Samples were shipped on dry ice to the Complutense University of Madrid (Spain), where they were stored at −80°C until an aliquot was sent on dry ice to Rutgers University (USA) for analyses. Human milk samples from Puerto Rico, the U.S. mainland, and Tanzania were collected either by hand expression or using the mother’s own sterile pump. Samples were frozen at the participants’ homes and transported on ice to the laboratory, where they were stored at −80°C until analysis.

### 2.2 DNA isolation, amplification and sequencing

The samples (1 mL) were centrifuged (15,000×*g* for 10 min at 4°C), and the fat layer was removed using a sterile swab. This step was repeated twice more to remove all fat. Then, the pellets together with a 200 μL fraction of the supernatants, were used for total DNA extraction employing the Dneasy PowerSoil Pro Kit (QIAGEN, Hilden, Germany), following the manufacturer’s instructions.

### 2.3 16S rRNA gene sequencing

Primers 515 F (5’-GTGYCAGCMGCCGCGGTAA-3′) and 806R (5′-GGACTACNVGGGTWTCTAAT-3′) were used to amplify the V4 hypervariable region of the bacterial 16S rRNA gene following the protocols for the Earth Microbiome Project.^1^ The concentration of the pooled, purified and barcoded DNA amplicons was determined using the Qubit dsDNA HS assay kit (Thermo Fisher, Waltham, MA, USA). Amplicons were sequenced at Genewiz, LLC. (South Plainfield, NJ, USA) using the Illumina MiSeq platform (Illumina, CA, USA) with the Illumina MiSeq 2 × 150 bp paired-end protocol (Illumina Inc., San Diego, CA, USA) using the Illumina MiSeq platform.

### 2.4 ITS sequencing

For fungi, the Internal Transcribed Spacer 2 (ITS2) region was amplified from DNA obtained from extracted milk samples with a single ITS3 forward primer (5′-AATGATACGGCGACCACCGAG ATCTACACTATGGTAATTGTGCATCGATGAAGAACGCAGC-3′) and a barcoded ITS4 reverse primer (5′-CAAGCAGAAGACGGCATA CGAGATTCCCTTGTCTCCAGTCAGTCAGCCTCCTCCGCTTAT TGATATGC-3′) (38). Primers included Illumina adapters and the reverse primer included a unique 12 base Golay barcode (XX) for pooled demultiplexing (5′-CAAGCAGAAGACGGCATACGAG ATXXXXXXXXXXXXCGGCTGCGTTCTTCATCGATGC-3′). To determine PCR cycle counts appropriate for the fraction of fungal material, qPCR was performed on 518 extracted human milk samples. For samples with a Ct value less than 30, 25 cycles of PCR was performed. For samples with a Ct value between 30–31, 33 cycles of PCR was performed; the higher cycle number was needed to ensure there was enough fungal material for sequencing. Samples with a Ct value above 33 were considered to contain no fungal material (matching a no-template control). Samples were purified by SPRI, pooled, and sequenced on the Illumina MiSeq platform using the Illumina MiSeq Reagent v3 600-cycle (2 × 300 bp).

### 2.5 qPCR detection of methanogen

For the detection of Methanogen from the samples, we performed qPCR to detect the copy number of Methanobacteriales with specific primers (Fwd: 5’-AGGAATTGGCGGGGGAGCAC-3′, Rev.: 5′-TGGGTCTCGCTCGTTG-3′) targeting the 16S rRNA gene fragment between positions 915 and 1,100 (48, 49). First, we use a universal primer (Fwd: 5′-ACTCCTACGGGAGGCAGCAG-3′, Rev.: 5′-ATTACCGCGGCTGCTGG-3′) targeting the 16S rRNA gene between position 314 and 540 (50) to detect the number of total bacteria and archaea. Part of the *E. coli* 16S gene fragment (NR_112558) was synthesized and diluted to 10^7^, 10^6^, 10^5^, 10^4^, 10^3^, 10^2^, and 10 copies as standards. The PCR program for total bacteria is initial at 95°C for 5 min, followed by 45 cycles of 10 s at 95°C, 10 s at 60°C, and 10 s at 72°C. Only the samples that had total bacterial copy numbers > 10^5^ were used to detect Methanogens. The standards for Methanogen were synthesized from part of the *Methanobacterium espanolae* 16S rRNA gene (NR_104983.1) and diluted to range from 10^7^ to 10 copies. The PCR program for Methanogen detection started with an initial step at 95°C for 10 min, followed by 40 cycles of 10 s at 95°C, 30 s at 57°C, and 20 s at 72°C. Both PCR was performed using a Quantstudio 3 system (Thermo Fisher, Waltham, MA, USA) with a Quantinova SYBR green PCR kit (Qiagen, Hilden, Germany) and 1uL of extracted DNA was used as the template. To avoid extrapolating beyond the standard curve, we set the detection limit at 10 copies for both PCR experiments.

### 2.6 Data analyses

Raw reads were demultiplexed and quality-filtered using the QIIME2 pipeline (v2022.2) (51). The quality-filtered reads were denoised and concatenated with DADA2 (52). Taxonomy was assigned to Amplicon sequence variants (ASVs) against the SILVA database v138.1 (53), using in QIIME 2 (q2-feature-classifier). The phylogenetic tree was generated by the FastTree algorithm (54). Negative control samples were also sequenced, and ASVs identified as contaminated by decontam (55) were removed from further analysis. The reads count was rarefied to 2,857 reads per sample (21 samples were excluded, Supplementary Table 1). The ASVs determined as contamination are listed in Supplementary Table 2.

Faith’s Phylogenetic diversity, observed ASV, and Shannon Index were calculated as alpha diversity metrics. The difference in alpha diversity between countries or continents was tested using the Kruskal-Wallis group test and with FDR *p*-value adjustment.

Jaccard, Bray Curtis, unweighted and weighted Unifrac distances were calculated to obtain pairwise beta-diversity, and dimensionality reduction on the distances was performed using Principal Coordinates Analysis (PCoA) method (ape v5.8). All alpha and beta diversity metrics are calculated on ASV level abundance. Permutational multivariate analysis of variance (PERMANOVA) was used to test the significance of different groups with 999 permutations (vegan v2.6-8).

Differentiated taxa were detected with ANCOMBC (2.6.0) (56) with Holm–Bonferroni correction, with a default prevalence cutoff of 10%. The shared taxa between countries were shown as an upset plot by UpSetR (1.4.0). The co-abundance network was performed on the genus level with taxa with abundance > 0.01 and prevalence > 10% using the SparCC method (57) (SpiecEasi v1.1.3), and the correlation cutoff was set to ± 0.3.

## 3 Results

### 3.1 Human milk bacteriome

The 16S rRNA gene (V4 region) sequencing analysis of the 497 milk samples included in this work (Supplementary Table 3) yielded 10,767,189 high-quality filtered sequences in total, ranging from 18 to 76,462 reads per sample [mean = 20,786 reads per sample; median (IQR) = 18,069 (9,542–28,173) sequences per sample]. The samples were rarefied to 2,857 sequences per sample (Supplementary Figure 1).

**Figure 1:**
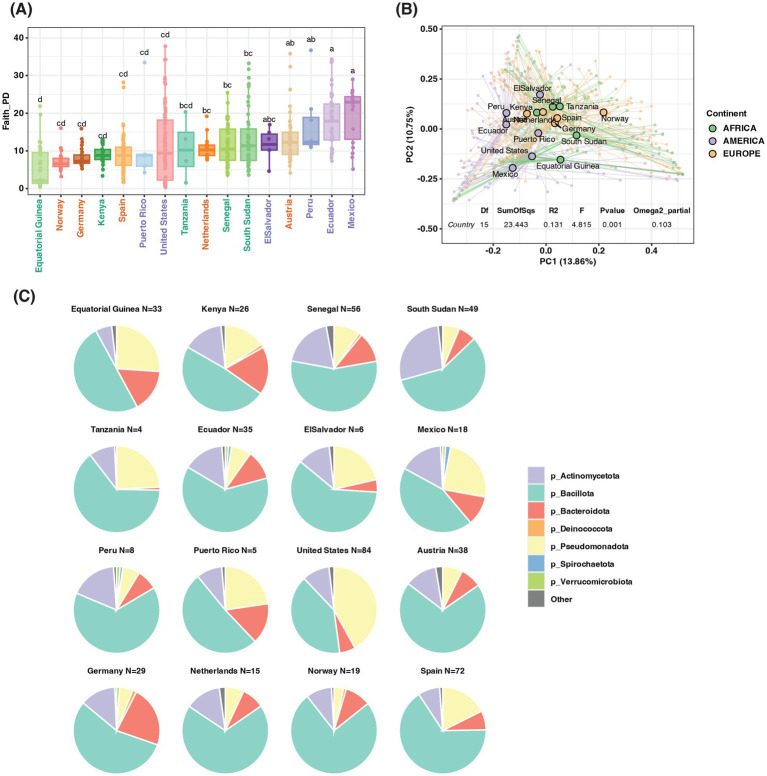
The bacterial difference between countries. **(A)** Alpha diversity in Faith PD, country ordered by median of Faith PD from low to high, Different letters show significant differences (Kruskal-Wallis test with FDR adjustment, *p* < 0.05). **(B)** PCoA plot based on Bray Curtis distance, the center of each country is in large dots, and individual samples are in small dots. PERMANOVA test of country effect is listed below. **(C)** The top abundant bacterial Phyla by country.

First, we applied a linear model to analyze the effect of country as well as some potential cofounders like maternal age, maternal body weight, birth mode, and the postpartum days of milk collection on the alpha diversity. Only the country showed a significant effect on the milk alpha diversity, and the effect size is bigger than the other variables (Supplementary Table 4). Next, we only focused on the difference between countries. As assessed by using the Faith PD diversity index, the milk alpha diversity oscillated between Equatorial Guinea and Mexico (Figure 1A); the same range between Equatorial Guinea (lowest) and Mexico (highest) in Observed ASVs and Shannon index (Supplementary Figure 2). Overall, there was a significant difference in alpha diversity between different countries. Among all the pairwise comparisons, Ecuador and Mexico had significantly higher Faith PD diversity than Germany, Norway, Spain, the United States, and Equatorial Guinea, Equatorial Guinea had significantly lower alpha diversity than Peru, Senegal, South Sudan, Austria, Mexico, and Ecuador. Most other pairwise comparisons were not significant after adjustment for multiple comparisons. Similar trends are shown in Observed ASVs, but not in Pielou evenness or Shannon index (Supplementary Table 5). When aggregated into the Continent level, America showed significantly higher alpha diversity in Faith PD and richness (observed ASVs), but metrics accounting for evenness (Pielou and Shannon index) showed higher evenness in Africa (Supplementary Figure 3).

For beta diversity, we also included all potential cofounders in the first PERMANOVA analysis and found that only country showed a significant effect on beta diversity (Supplementary Table 6). Therefore, in the following analysis, we only included the country or continent. We detected a significant effect by country with the omega square effect size is 0.103 (meaning about 10.3% of the variation is from the country effect; *p*-value = 0.001). Mexico, Equatorial Guinea, and Norway showed the most segregated centroids in PCoA analysis based on Bray Curtis distance (Figure 1B). Some European and African countries were close to each other in the PCoA plot, including Spain, Germany, Austria, Kenya, Senegal, and Tanzania, indicating similar bacterial structures. The countries from different continents also differ in PC1, the countries from America all had negative PC1 values while most of the rest countries had positive PC1 values. Jaccard and Unifrac distance showed a similar effect, but the effect size of weighted Unifrac (0.120) is bigger than Jaccard (0.068) or unweighted Unifrac (0.064), suggesting the difference between countries was due to abundant taxa (Supplementary Figure 4). The effect of the continent on beta diversity was also significant but had a smaller effect size (omega square range from 0.022 to 0.043), and based on Bray Curtis and Jaccard, the distance of the center of samples from Europe and Africa was closer than the distance to America (Supplementary Figure 5).

**Figure 2:**
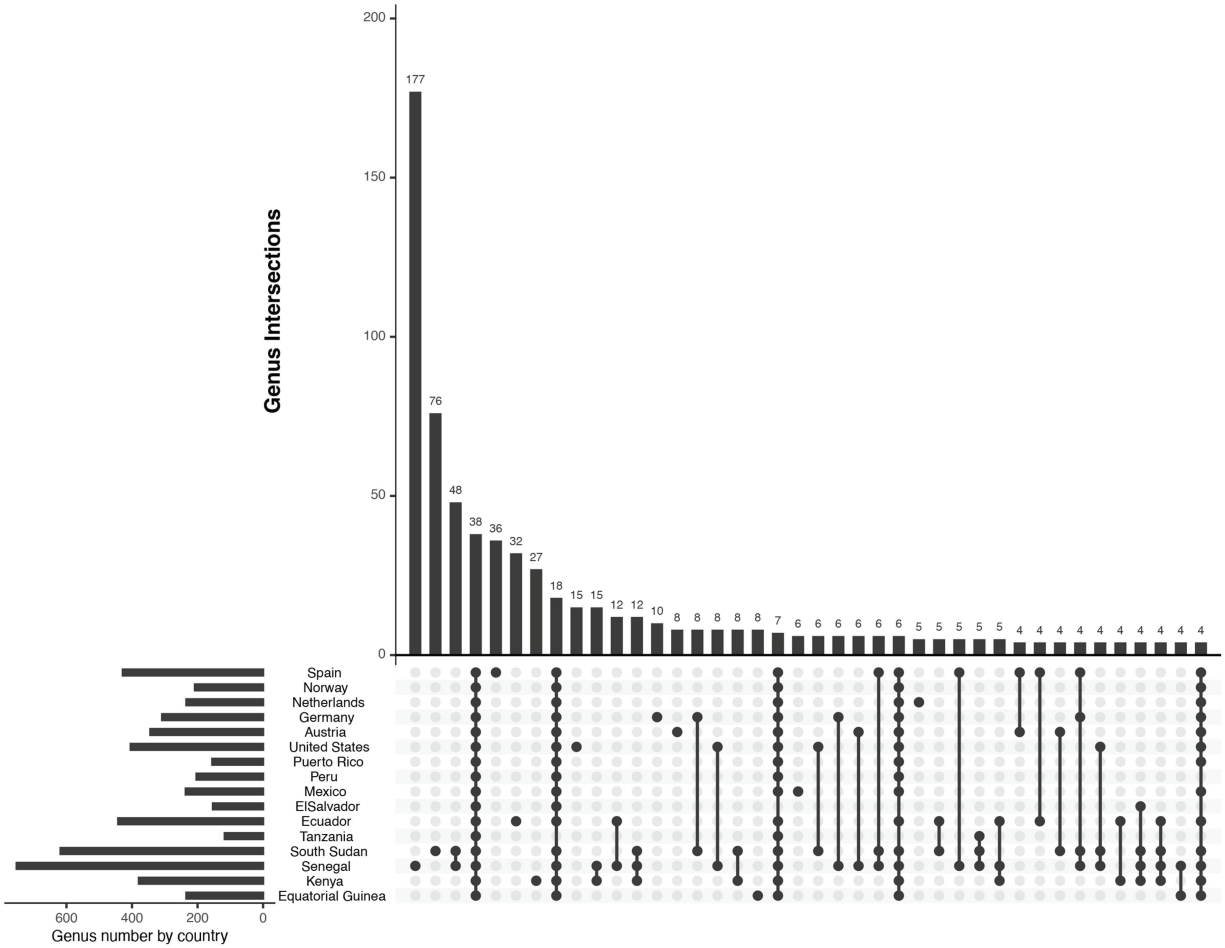
Upset plot of unique or shared genera between countries.

In total 14,581 clean ASVs were generated from 16S sequencing, belonging to 41 phyla, 1,333 genera and 2,774 species. Most of them corresponded to seven major phyla: Bacillota, Pseudomonadota, Actinomycetota, Bacteroidota, Spirochaetota, Verrucomicrobiota, and Deinococcota, (Figure 1C). Among the 1,333 genera detected in this work, only 38 of them were found in samples from all the countries (Figure 2), while 18 were shared by all countries except for those from Tanzania and 7 were shared by all countries except for those from El Salvador. The genera with the top three highest frequency of detection were *Staphylococcus* (99.2% of samples; present in samples from all countries), *Streptococcus* (97.4% of samples; present in all countries), and *Bifidobacterium* (93.2% of samples; present in all countries). Overall, the top three most abundant genera were the same as the top three prevalent genera, *Streptococcus* (mean abundance 23%), *Staphylococcus* (mean abundance 16.8%), and *Bifidobacterium* (mean abundance 5.9%). Other highly abundant (> 1%) and universal genera include *Pseudomonas*, *Corynebacterium*, *Acinetobacter*, *Rothia*, *Lactobacillus*, *Prevotella*, *Bacteroides*, and unclassified genera from Muribaculaceae, Enterobacteriaceae, and Lachnospiraceae (Supplementary Table 7).

**Figure 3:**
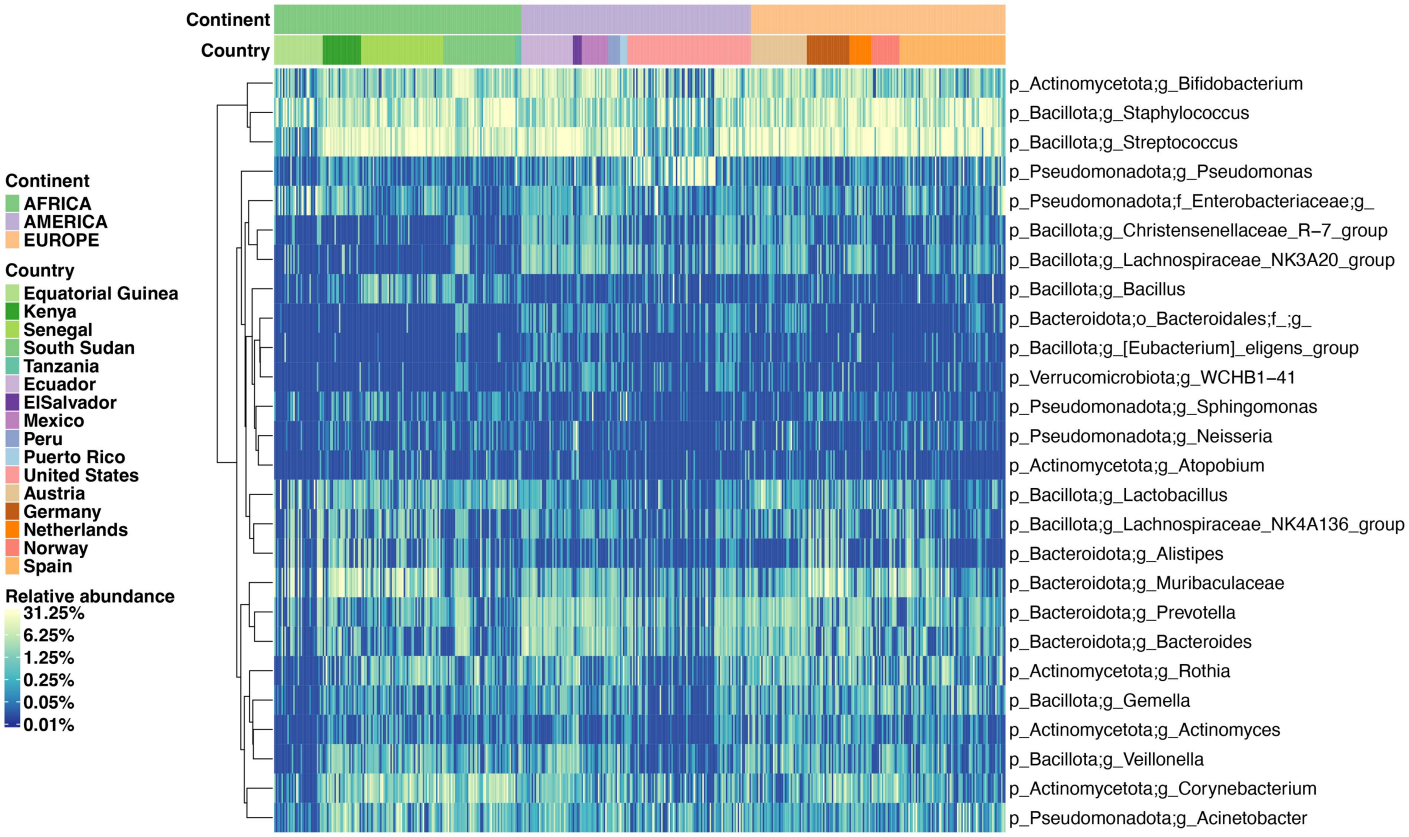
Supervised heatmap of genera relative abundance significantly different between countries. The differentiated genera were detected by ANCOM global comparison with Holm–Bonferroni correction with adjusted *p*-value < 0.05.

Samples from mainland USA are enriched in *Pseudomonas*, and depleted in *Streptococcus*, and *Lactobacillus*. Mexico and Ecuador are enriched in *Bifidobacterium*, *Bacteroides,* and *Prevotella*. Many countries from the European cohort are depleted in *Bacillus* and *Alistipes* (except Germany), enriched in *Staphylococcus*. Many countries from the African cohort are enriched in *Alistipes* and *Bacillus* and depleted in *Bacteroides* and *Gemella*. Kenya enriched in *Acinetobacter*, *Alistipes*, *Lactobacillus,* and Muribaculaceae (Figure 3 and Supplementary Figure 6).

**Figure 4:**
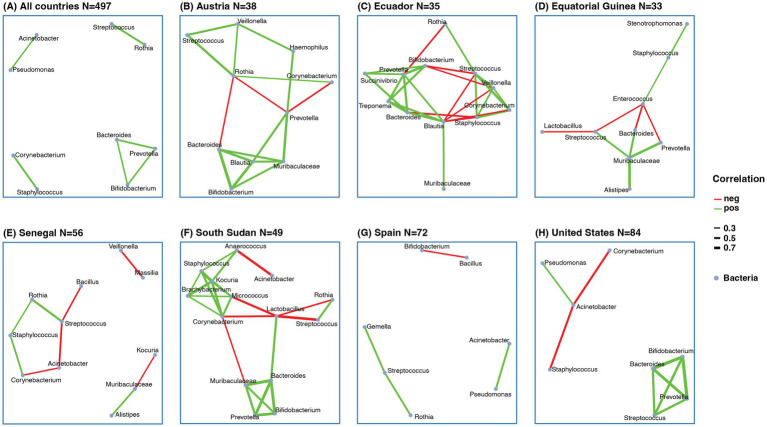
Co-abundance network of human milk bacteria. **(A)** Co-abundance network with samples from all countries. Co-abundance network from individual country, **(B)** Austria, **(C)** Ecuador, **(D)** Equatorial Guinea, **(E)** Senegal, **(F)** South Sudan, **(G)** Spain, **(H)** United States. The correlation was calculated based on sparCC with abundance of genera > 0.01 and prevalence > 10%. Only correlations > 0.3 or <−0.3 and pseudo-*p*-value < 0.05 were selected. The red line indicated negative correlation, the green line indicated positive correlation, line width indicated correlation value.

Co-abundance network analysis of human milk bacteria only showed positive associations, particularly *Bacteroides*, *Prevotella*, and *Bifidobacterium* formed positive connections between each other. Other positive connections are between *Staphylococcus* and *Corynebacterium*; *Streptococcus* and *Rothia*; *Pseudomonas* and *Acinetobacter* (Figure 4A). That different bacterial genera occupied central positions, indicates country-specific structures and ecological roles within the microbial communities. For example, in Austria, *Blautia* and *Rothia* served as central nodes with strong associations to multiple genera. In South Sudan, *Lactobacillus*, *Corynebacterium*, and *Kocuria* were the most highly connected, while in Ecuador, *Streptococcus*, *Veillonella*, *Staphylococcus*, *Blautia*, *Treponema*, *Bifidobacterium*, and *Prevotella* showed the highest connectivity. These variations likely reflect underlying differences in genus abundance. Indeed, genera such as *Bifidobacterium*, *Staphylococcus*, *Streptococcus*, *Lactobacillus*, *Corynebacterium*, *Rothia*, *Bacteroides*, *Prevotella*, and *Pseudomonas* were identified as differentially abundant and consistently appeared as key hubs in their respective networks. Notably, the global network combining all countries’ data was the most robust and densely interconnected, highlighting overarching co-abundance patterns across diverse populations.

Despite these differences, we also observed some recurring correlation patterns across countries, similar to those in the global network. For instance, connections among *Bacteroides*, *Prevotella*, and *Bifidobacterium* were seen in Austria, Ecuador, South Sudan, and the United States. However, specific patterns varied: in Austria, *Blautia* and *Muribaculaceae* were also connected (Figure 4B); in Ecuador, *Blautia*, *Succinivibrio*, *Treponema*, and *Bacteroides* formed key links (Figure 4C); in South Sudan, *Muribaculaceae* showed strong associations (Figure 4F); and in the United States, *Streptococcus* emerged as a connected node (Figure 4H). Networks in Austria, Ecuador, Equatorial Guinea, and South Sudan exhibited greater complexity and connectivity compared to others.

### 3.2 Detection of total bacteria methanogenic archaea

Based on 16S sequencing, we were able to detect three genera from Methanobacteriales (*Methanobrevibacter* (0–2.6%), *Methanosphaera* (0–16.7%), and *Methanobacterium* (0–33.3%) Supplementary Table 8).

We also applied a standard curve qPCR method to detect Methanobacteriales. Based on our 16S rRNA sequencing results, which showed that the methanogen relative abundance ranged from 0.03 to 1% in samples where methanogen reads were detected. Given these proportions, if a sample contained fewer than 10⁵ total bacterial cells, the number of methanogen cells could be as low as 30 copies -close to the detection limit-. Therefore, we performed methanogen qPCR only on samples with a total bacterial cell count exceeding 10⁵. When examining a subset of the samples (268 samples from 7 countries) with qPCR, we found that only 21 (8%) had total bacteria/archaea 16S gene copy number >10^5^ per 1 μL DNA, evidencing the low bacterial density in these human milk samples from healthy women. Furthermore, this threshold of detection of 10^5^ gene copies found in our study likely overestimates the number of bacterial cells in the samples, since bacteria can have multiple 16S gene copies. Among the 21 samples, only two, one from South Sudan and the other from Equatorial Guinea had detectable methanogenic archaea (Supplementary Table 9).

### 3.3 Human milk mycobiome

**Figure 5:**
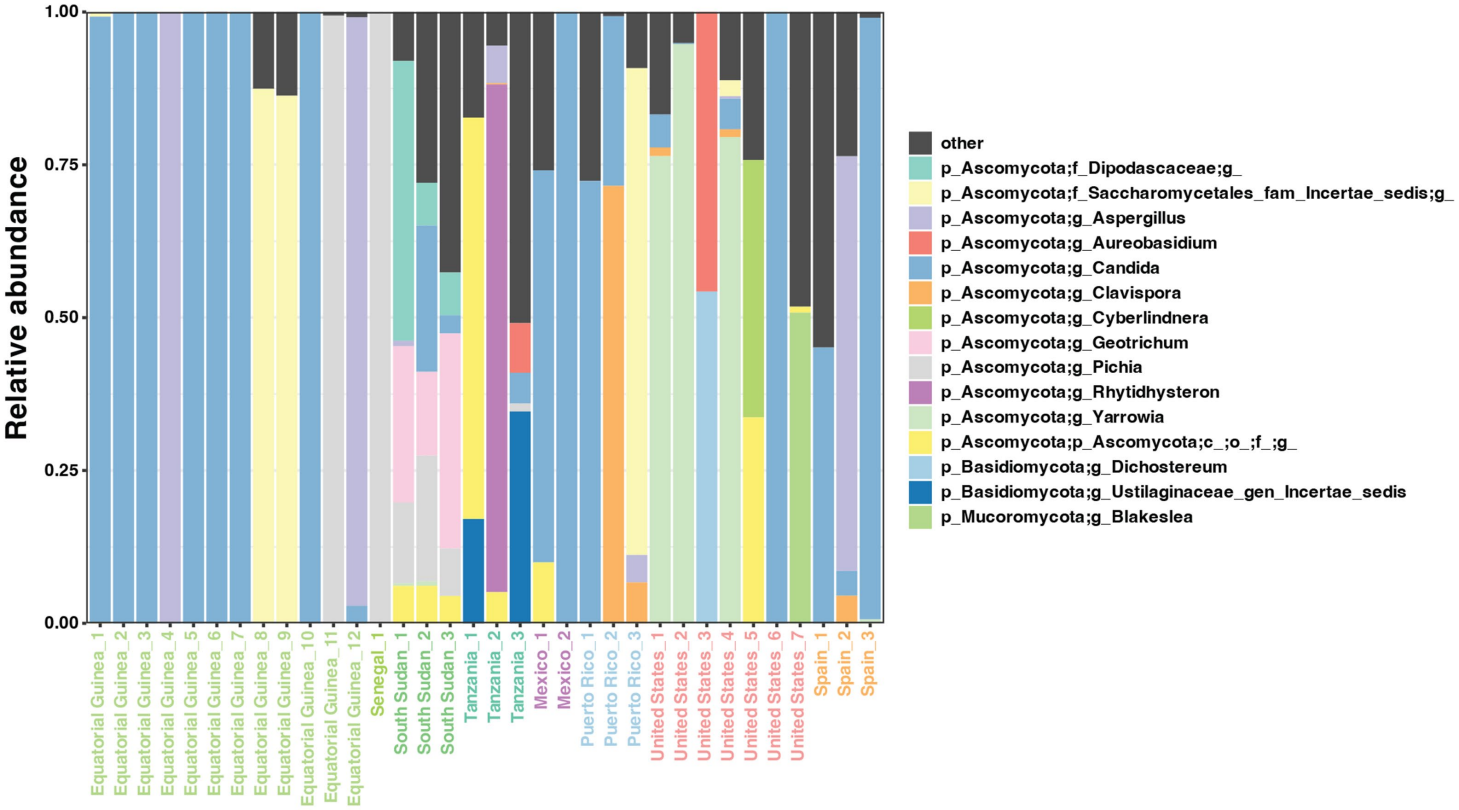
Relative abundance of ITS2-based major fungi genera in 34 samples.

The presence of fungi was evaluated in the whole collection of 518 samples (Supplementary Table 10). Only 6.6% of samples (*n* = 34) were positive based on ITS2 amplification and they belonged to the following cohorts: Equatorial Guinea (*n* = 12, 36.4%), Senegal (*n* = 1, 1.7%), South Sudan (*n* = 3, 5.7%), Tanzania (*n* = 3, 75%), Mexico (*n* = 2, 11.1%), mainland US (*n* = 7, 8.2%), Puerto Rico (*n* = 3, 60%), and Spain (*n* = 3, 3.9%). Among the fungi-positive samples, three fungal phyla (Ascomycota, Basidiomycota, and Mucoromycota) and 12 major genera were detected, dominated by *Candida* (Figure 4). Some of the fungal genera, such as *Candida,* and *Malassezia,* are frequent inhabitants of the human skin while others, such as *Dichostereum or Blakeslea*, are typically associated other to air, soil, and decaying plant matter, suggesting environmental or laboratory contamination of milk samples (Figure 5, Supplementary Table 11).

### 3.4 Interkingdom co-abundance networks

**Figure 6:**
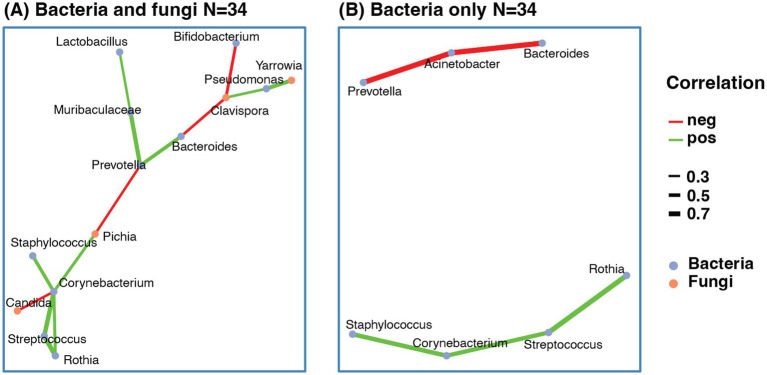
Co-abundance network of human milk microbes. **(A)** Co-abundance network with both bacteria and fungi. **(B)** Co-abundance network with only bacteria using the same sample as the interkingdom network. The correlation was calculated based on sparCC with abundance of genera > 0.01, and prevalence > 10%. Only correlations > 0.3 or <−0.3 and pseudo-*p*-value < 0.05 were selected. The red line indicated negative correlation, the green line indicated positive correlation, line width indicated correlation value.

The interkingdom co-abundance network showed that *Prevotella*, *Corynebacterium* and *Clavispora* were the centers of the network (Figure 6A). Besides *Clavispora*, there were three fungal genera involved in the network, including *Candida*, *Pichia*, and *Yarrowia*. Most bacterial genera involved were the common genera found in the bacteria-only networks, including *Bacteroides*, *Prevotella*, Bifidobacterium, *Staphylococcus*, *Corynebacterium*, *Streptococcus*, *Pseudomonas*, *Lactobacillus*, and *Rothia*. Compared with the bacteria-only network with all samples (Figure 4A) or the same sample size as the interkingdom network (Figure 6B), the bacteria correlations remained similar, and the fungal genera acted as “bridges” connecting the isolated parts of the bacteria-only network.

## 4 Discussion

Human milk poses some challenges for microbiome studies since the procedures used for milk and milk DNA processing and analysis, its low biomass in healthy women (as observed in this work by qPCR), and the risk of skin and environmental contamination during sampling may also be a cause of artifacts or biases in these studies (40, 58). In this work, the application of the same methodology, from DNA extraction to data analysis, to a large collection of milk samples from 16 countries confirmed the existence of large inter-individual and inter-cohort variations in the composition of the human milk bacteriome and mycobiome, as described previously (22, 30, 38, 39, 59). The lack of clear separation between milk samples from women in different geographical regions may be due to the high intraindividual variability among milk samples from the same continent or country (40). Our findings align with previous studies focused on the bacteriome of human milk samples worldwide, particularly with those obtained within the framework of the INSPIRE study (30, 40). *Staphylococcus* and *Streptococcus* were the dominant genera by prevalence and abundance, detected in 99.2 and 97.4% of our samples, closely matching the 98.7 and 97.7% reported in INSPIRE (30), and consistent with other studies (22).

Similarly, the INSPIRE study reported a high abundance of *Streptococcus* in Peruvian and *Pseudomonas* in U.S. samples (30), consistent with our findings. Both studies also observed a higher relative abundance of Pseudomonadota (formerly *Proteobacteria*) in African versus European cohorts. However, as in INSPIRE, additional core taxa varied across cohorts. In our study, other frequent genera included *Bifidobacterium*, *Pseudomonas*, *Corynebacterium*, *Acinetobacter*, *Rothia*, *Lactobacillus*, *Prevotella*, and *Bacteroides*, with no clear geographical differences. Unlike INSPIRE, we did not analyze infant fecal samples, limiting our ability to assess the role of milk microbiota in seeding the infant gut. INSPIRE’s comparison of milk and infant feces provided evidence of this relationship (30). Future studies are needed to further explore vertical transmission through breastfeeding.

In this study, potential intrakingdom and interkingdom relationships were investigated through the establishment of co-abundance networks of bacteria and bacteria-fungi, respectively. In relation to intrakingdom associations, there was a positive correlation between the presence of *Staphylococcus* and *Corynebacterium*. The same observation has been performed repeatedly when studying other human niches and, particularly, the skin (60–62). It is noteworthy that mammary glands are highly specialized organs, likely evolving from ancestral cutaneous apocrine-like glands (63). The populations of *Staphylococcus* and *Corynebacterium* appear to respond synchronously, both in health and disease, to shared host or environmental factors within the mammary ecosystem (64). This synchrony is also observed in various skin regions, including the nasal sinuses and ocular surfaces and glands (62, 65–68). Additionally, skin secretions and milk share common components such as exfoliated epithelial cells and nutrients, including urea, amino acids, peptides, glycoproteins, glycerol, phospholipids, and others. Coagulase-negative staphylococci utilize amino acids mainly provided by their own proteolytic activities and, in turn, corynebacteria need these same amino acids and are cross-fed by their skin partners (69). The proteolytic properties of resident staphylococci also have a protective role for corynebacteria since they are able to inactivate antibacterial proteins and peptides (69, 70). In addition, lipophilic corynebacteria lack fatty acid synthase and, consequently, are fatty acid auxotrophs (71). Interestingly, it seems that *Staphylococcus epidermidis* and *Corynebacterium* spp. use different glycans as molecular decoys for binding to human skin and sweat (72). More specifically, sialic acid and fucose, which are also key components of human milk oligosaccharides, are binding epitopes for *staphylococci* while *N*-glycans did not provide binding epitopes for *Corynebacterium*, consistent with a lack of competition between them for these substrates.

A positive correlation was also observed among *Bacteroides*, *Prevotella*, and *Bifidobacterium*. DNA from these three genera has already been detected in human milk (15, 30, 73–75), and they are common inhabitants of the human gut (76). Their abundance is higher in children than in adults (77) and is reduced in caesarean-delivered infants in comparison with vaginally-delivered infants (78). These three genera have been proposed as biomarkers of diet and lifestyle (79). They may form metabolic networks in the infant gut, playing synergistic and complementary roles. For example, certain *Bifidobacterium* species are well adapted to the human milk and infant gut environment due to their ability to metabolize human milk oligosaccharides (HMOs) (80). In the process, they produce lactic and acetic acids, which promote the growth of short-chain fatty acid (SCFA) producers like *Prevotella* and *Bacteroides*. These species further enhance glucose metabolism and generate SCFAs and vitamins that support health (81). During lactation, *Prevotella* and *Bacteroides* also metabolize milk-derived amino acids (82), and later become key to breaking down complex polysaccharides once solid foods are introduced. Thus, human milk may seed the infant gut with bacteria that support gut health both early and later in life.

Supporting this idea, a fecal microbiota transplant (FMT) study in patients with autism spectrum disorder (ASD) found that 2 years post-treatment, the gut microbiome was dominated by *Bacteroides*, *Prevotella*, and *Bifidobacterium*, alongside a reduction in ASD-associated taxa (82), Similarly, these genera are often depleted in individuals colonized by *Clostridioides difficile* (83), further highlighting their potential health benefits.

In this study, *Bifidobacterium* was negatively associated with *Pseudomonas*, consistent with previous results showing the antagonistic activity of bifidobacteria against some species of *Pseudomonas* and, particularly, against *Pseudomonas aeruginosa* (84–86). In the same direction, pretreatment of human corneal epithelial cells with a strain of *Bifidobacterium longum* subsp. *infantis* protected them from infection by a *P. aeruginosa* strain (87). Evidence suggests that the protection against *P. aeruginosa* cellular infection is through modulation of the expression of IL-8 and beta-defensin-2 (88). Indeed, this protection has been harnessed to slow the decay of the microbiological quality caused by *P. aeruginosa* by *B. longum* subsp. *infantis* biofilms in the inner surface of cheese packages (89). Therefore, it seems that bifidobacteria displays, to some extent, mechanisms of competitive exclusion against pseudomonas.

In a study including 80 mothers from four countries [Finland, Spain, South Africa, and China; (28)], in another of 65 mothers from Spain (32), detection of the fungi ranged from 35 to 86%. In a Canadian cohort with 271 mothers, it was lower, 21% of the human milk samples (38), but still higher than the 7% in the current study (ranging from 2 to 36%), for reasons that are not clear. This could be due to methodological issues, or to higher density of bacteria that inhibit fungi. Interactions between bacteria and fungi are poorly known although they may be relevant for health (90, 91). Two major negative correlations were detected in our study, one between *Bifidobacterium* and *Clavispora*, and the second between *Corynebacterium* and *Candida*. These results are consistent with those in a recent study reporting negative correlation between *Bifidobacterium* and *C. albicans* in the milk of mothers who delivered vaginally (39). A body of evidence suggests that in the gut, bifidobacteria provide resistance against colonization by yeasts, particularly by *Candida albicans* (92–96). More recently, another study in children and adolescents reported negative correlation between *Bifidobacterium* and *Candida*, with higher *Candida* low *Bifidobacterium* associated with depression (97). Other studies have not found fungal-bacterial interactions in the feces of healthy subjects (98). As for *Corynebacterium* and *Candida*, it is long known their negative association and the protective effect of the former against infections (99–101). Interestingly, high oral *C. albicans* and low *Corynebacterium* appears to be a signature of oral carcinoma and head and neck cancer (102).

The qPCR analysis detected the presence of detectable methanogenic archaea (Methanobacteriales) in two African samples, one from South Sudan and one from Equatorial Guinea. Consistent with the qPCR results, the methanogenic archaea detection rate was higher in African and American countries based on 16S sequencing. However, the detection rate of 16S sequencing is much higher than that of qPCR, possibly because the DNA concentration in the milk sample is low, and the 1 μL of input DNA used for qPCR may not be sufficient to detect the methanogenic archaea. Archaea are among the neglected microbes in human microbiome studies because of technical challenges in detection (103), although their presence has been previously reported in human colostrum and milk (31, 104), and in the gut of babies (105–107). Archaeal DNA has been detected in human milk, albeit at low frequency and abundance. Togo et al. (31) identified *Methanobrevibacter smithii* in approximately 25% of colostrum and milk samples using species-specific qPCR, whereas *Methanobrevibacter oralis* was not detected. Another study reported the presence of DNA from *Methanoculleus*, *Methanosarcina*, and *Methanobrevibacter* in Mexican mother-infant dyads, suggesting that colostrum may serve as a source of neonatal archaea (103). Similarly, Grine et al. (105) proposed maternal transmission of *M. smithii*. Methanogenic archaea—including *Methanobrevibacter* spp., *Methanosphaera stadtmanae*, and members of the *Methanomassiliicoccales*—are recognized as consistent but low-abundance constituents of the infant gut microbiome (103, 106, 108). However, detection remains limited by methodological biases favoring bacterial over archaeal targets, such as inefficiencies in DNA extraction, primer design, and reference databases. Notably, a recent gut microbiome survey in Africa—where our archaeal-positive samples were most prevalent -did not assess archaeal DNA (109). To advance characterization of archaeal diversity and function, we recommend: (a) the use of archaeal-specific or dual-target primers and nested PCR; (b) shotgun metagenomics for unbiased genomic and functional profiling; (c) optimized DNA extraction protocols tailored to the resilient archaeal cell wall; (d) expansion of archaeal reference databases; (e) integration of multi-omics approaches to detect archaeal metabolites such as methane; and (f) improved cultivation methods using anaerobic conditions and archaeal-specific substrates.

This study has several limitations. There was a big variation in sample size between countries (from 4 to 85), which weakens the statistical power. Also, sample collection was not absolutely standardized, which may introduce a systematic bias between cohorts. Despite the limitations, this work highlights differences in the human milk bacterial and fungal microbiome across geographies, emphasizing the need for global studies that provide a better understanding of the human microbiome and the interkingdom relationships that may explain microbiota structures, with implications for maternal and infant health.

## Data Availability

The datasets presented in this study can be found in online repositories. The names of the repository/repositories and accession number(s) can be found at: https://www.ncbi.nlm.nih.gov/, PRJNA1216262.

## Footnotes

^1^ https://earthmicrobiome.org/protocols-and-standards/16s/

## Supplementary material

SUPPLEMENTARY FIGURE 1 Bacterial 16S rarefaction curve **(A)** and goods coverage of samples **(B)**. Rarefy depth set to 2857.

SUPPLEMENTARY FIGURE 2 Alpha diversity between countries. **(A)** Observed ASVs, **(B)** Pielou evenness, **(C)** Shannon index. Countries ordered by median of diversity from low to high, different letters show significant differences (Kruskal-Wallis test with FDR adjustment, *p* < 0.05).

SUPPLEMENTARY FIGURE 3 Alpha diversity by continent. **(A)** Faith PD, **(B)** observed ASVs, **(C)** Pielou evenness, **(D)** Shannon Index. Difference between continents tested by Kruskal-Wallis and with FDR adjustment. **p* < 0.05, ***p* < 0.01, ****p* < 0.001, *****p* < 0.0001.

SUPPLEMENTARY FIGURE 4 PCoA plots based on beta diversity in different countries. **(A)** Jaccard distance, **(B)** unweighted Unifrac distance, **(C)** weighted Unifrac distance. The center of each country is in large dots, and individual samples are in small dots. PERMANOVA test of country effect is listed below.

SUPPLEMENTARY FIGURE 5 PCoA plots based on beta diversity in different continents. **(A)** Bray Curtis distance, **(B)** Jaccard distance, **(C)** unweighted Unifrac distance, **(D)** weighted Unifrac distance. The center of each country is in large dots, and individual samples are in small dots. PERMANOVA test of country effect is listed below.

SUPPLEMENTARY FIGURE 6 Difference of relative abundance between countries of selected genera. Boxplot of the log-transformed relative abundance of differentiated genera selected by ANCOM. In each panel, the countries are ordered by the median abundance of the genus. Different letters show significant differences (Kruskal-Wallis test, adjusted with FDR, *p* < 0.05).
