# Supplementary figures and images for: The human milk bacteriome and mycobiome and their inter-kingdom interactions viewed across geography
Source: Front Nutr. 2025 Jul 7;12:1610346. doi: 10.3389/fnut.2025.1610346 (PMC12277152; doi:10.3389/fnut.2025.1610346)

**(A)**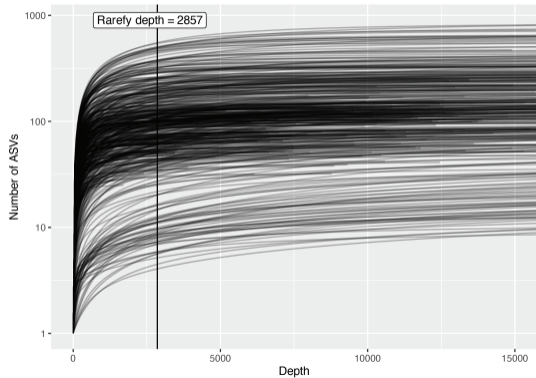**(B)**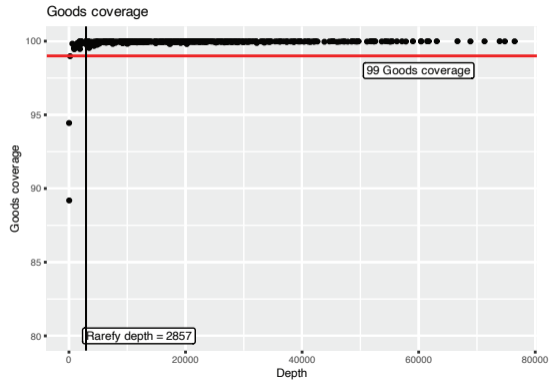

Supplement: SUPPLEMENTARY FIGURE 1 — Bacterial 16S rarefaction curve (A) and goods coverage of samples (B). Rarefy depth set to 2857. [file Image_1.pdf]

**(A)**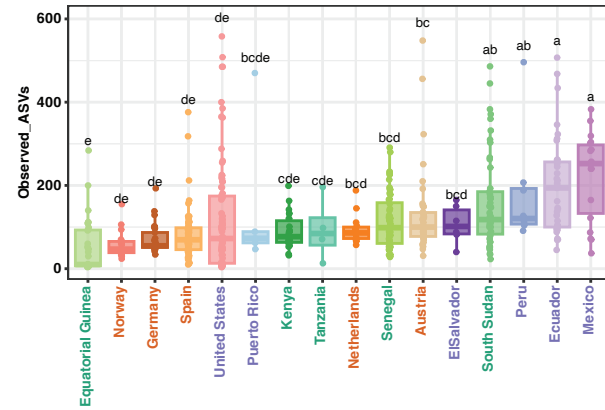**(B)**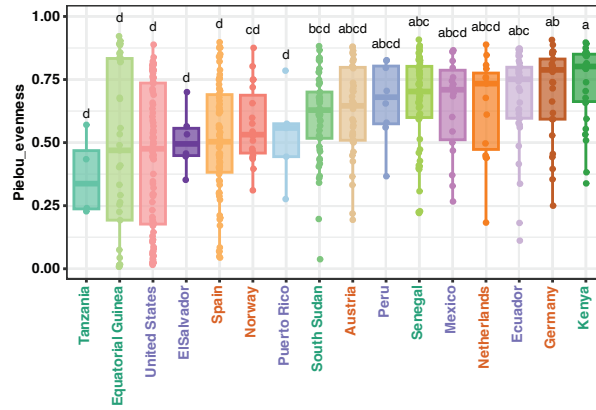**(C)**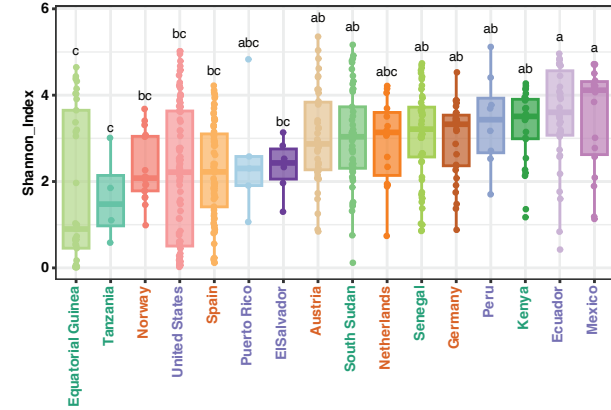

Supplement: SUPPLEMENTARY FIGURE 2 — Alpha diversity between countries. (A) Observed ASVs, (B) Pielou evenness, (C) Shannon index. Countries ordered by median of diversity from low to high, different letters show significant differences (Kruskal-Wallis test with FDR adjustment, p < 0.05). [file Image_2.pdf]

**(A)**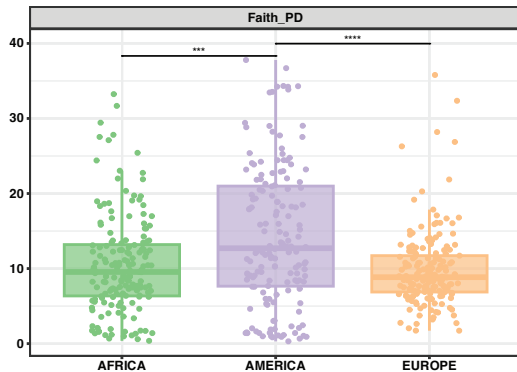**(B)**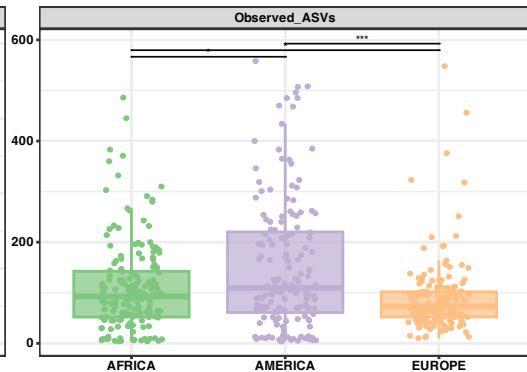**(C)**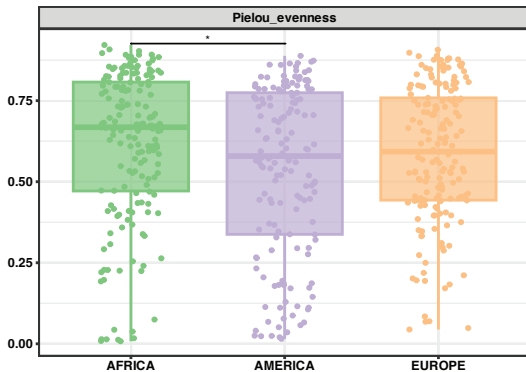**(D)**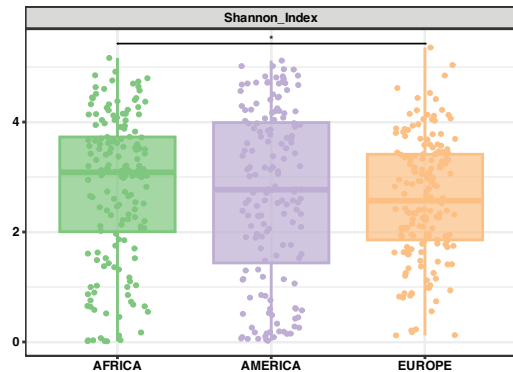

Supplement: SUPPLEMENTARY FIGURE 3 — Alpha diversity by continent. (A) Faith PD, (B) observed ASVs, (C) Pielou evenness, (D) Shannon Index. Difference between continents tested by Kruskal-Wallis and with FDR adjustment. *p < 0.05, **p < 0.01, ***p < 0.001, ****p < 0.0001. [file Image_3.pdf]

**(A) Jaccard**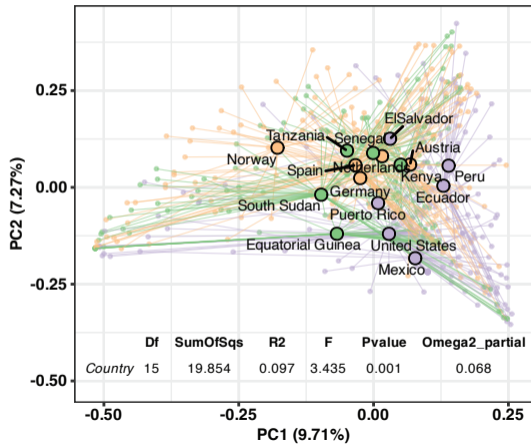**(B) Unweighted Unifrac**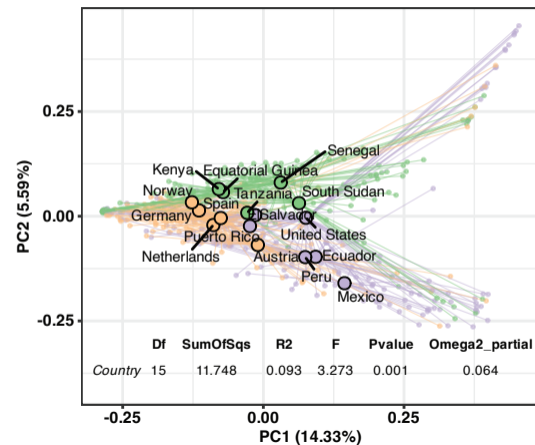**(C) Weighted Unifrac**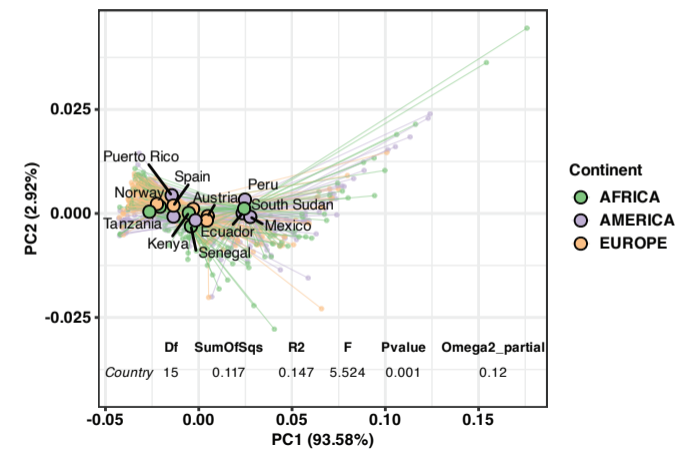

Supplement: SUPPLEMENTARY FIGURE 4 — PCoA plots based on beta diversity in different countries. (A) Jaccard distance, (B) unweighted Unifrac distance, (C) weighted Unifrac distance. The center of each country is in large dots, and individual samples are in small dots. PERMANOVA test of country effect is listed below. [file Image_4.pdf]
