# Supplementary material for: The human milk bacteriome and mycobiome and their inter-kingdom interactions viewed across geography
Source: Front Nutr. 2025 Jul 7;12:1610346. doi: 10.3389/fnut.2025.1610346 (PMC12277152; doi:10.3389/fnut.2025.1610346)
Supplement: SUPPLEMENTARY FIGURE 5 — PCoA plots based on beta diversity in different continents. (A) Bray Curtis distance, (B) Jaccard distance, (C) unweighted Unifrac distance, (D) weighted Unifrac distance. The center of each country is in large dots, and individual samples are in small dots. PERMANOVA test of country effect is listed below. [file Image_5.pdf]

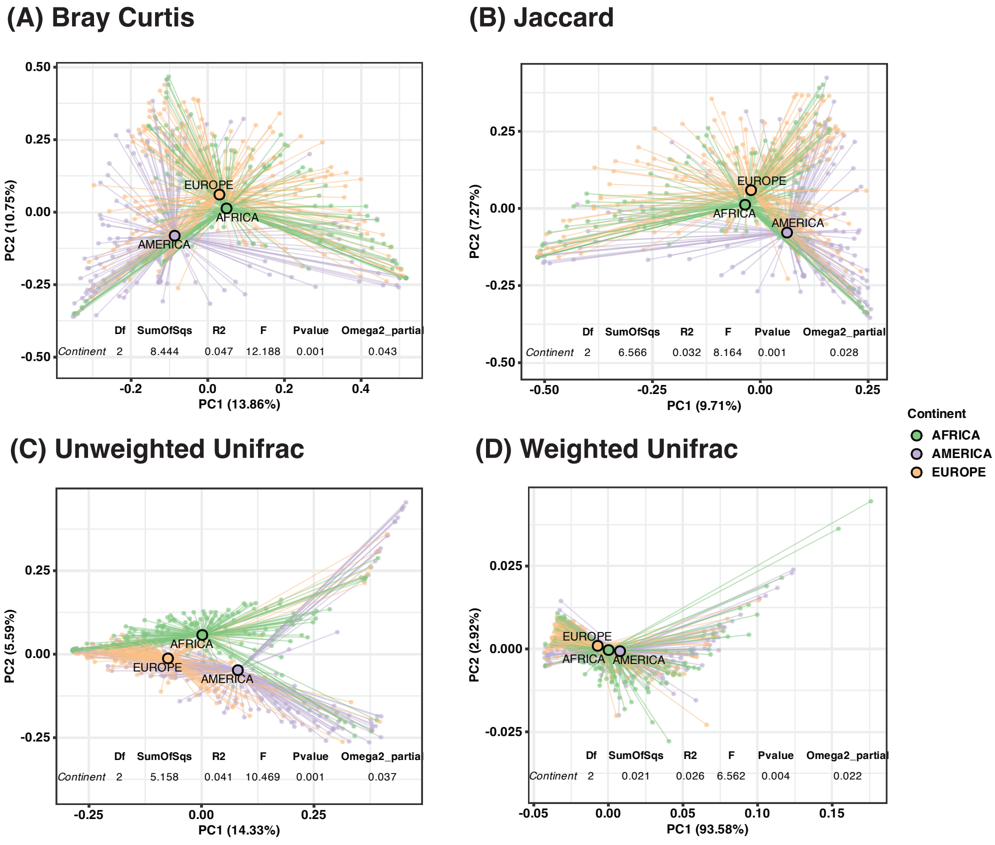

(A) Bray Curtis
PC1 (13.86%)
PC2 (10.75%)
EUROPE
AFRICA
AMERICA
Df SumOfSqs R2 F Pvalue Omega2_partial
Continent 2 8.444 0.047 12.188 0.001 0.043
(B) Jaccard
PC1 (9.71%)
PC2 (7.27%)
EUROPE
AFRICA
AMERICA
Df SumOfSqs R2 F Pvalue Omega2_partial
Continent 2 6.566 0.032 8.164 0.001 0.028
(C) Unweighted Unifrac
PC1 (14.33%)
PC2 (5.59%)
AFRICA
EUROPE
AMERICA
Df SumOfSqs R2 F Pvalue Omega2_partial
Continent 2 5.158 0.041 10.469 0.001 0.037
(D) Weighted Unifrac
PC1 (93.58%)
PC2 (2.92%)
EUROPE
AFRICA
AMERICA
Df SumOfSqs R2 F Pvalue Omega2_partial
Continent 2 0.021 0.026 6.562 0.004 0.022
Continent
AFRICA
AMERICA
EUROPE
