# Supplementary material for: The human milk bacteriome and mycobiome and their inter-kingdom interactions viewed across geography
Source: Front Nutr. 2025 Jul 7;12:1610346. doi: 10.3389/fnut.2025.1610346 (PMC12277152; doi:10.3389/fnut.2025.1610346)
Supplement: SUPPLEMENTARY FIGURE 6 — Difference of relative abundance between countries of selected genera. Boxplot of the log-transformed relative abundance of differentiated genera selected by ANCOM. In each panel, the countries are ordered by the median abundance of the genus. Different letters show significant differences (Kruskal-Wallis test, adjusted with FDR, p < 0.05). [file Image_6.pdf]

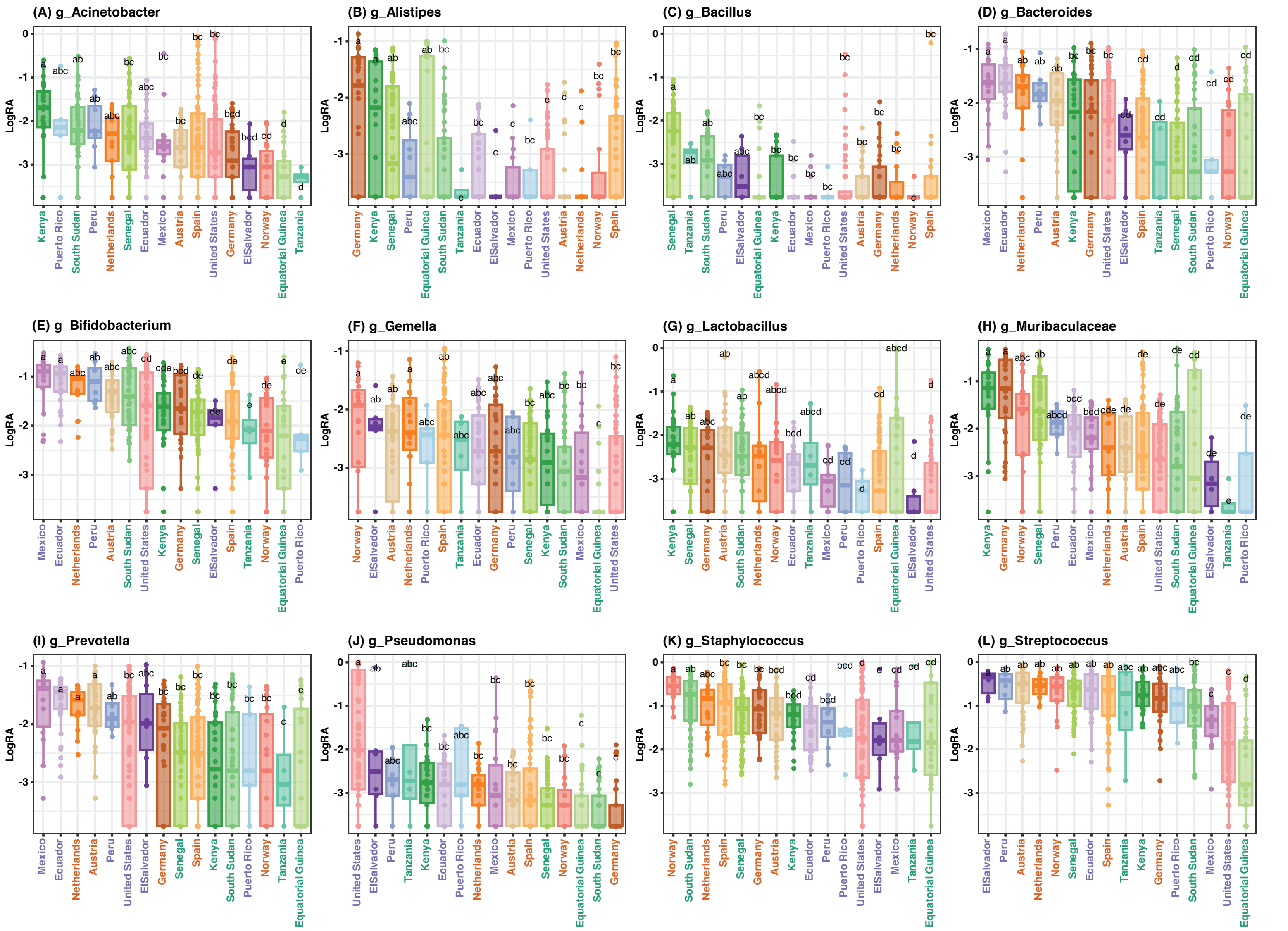
(A) g_Acinetobacter
LogRA
0
-1
-2
-3
Kenya
Puerto Rico
South Sudan
Peru
Netherlands
Senegal
Ecuador
Mexico
Austria
Spain
United States
Germany
ElSalvador
Norway
Equatorial Guinea
Tanzania
(B) g_Alistipes
LogRA
-1
-2
-3
Germany
Kenya
Senegal
Peru
Equatorial Guinea
South Sudan
Tanzania
Ecuador
ElSalvador
Mexico
Puerto Rico
United States
Austria
Netherlands
Norway
Spain
(C) g_Bacillus
LogRA
0
-1
-2
-3
Senegal
Tanzania
South Sudan
Peru
ElSalvador
Equatorial Guinea
Kenya
Ecuador
Mexico
Puerto Rico
United States
Austria
Germany
Netherlands
Norway
Spain
(D) g_Bacteroides
LogRA
-1
-2
-3
Mexico
Ecuador
Netherlands
Peru
Austria
Kenya
Germany
United States
ElSalvador
Spain
Tanzania
Senegal
South Sudan
Puerto Rico
Norway
Equatorial Guinea
(E) g_Bifidobacterium
LogRA
-1
-2
-3
Mexico
Ecuador
Netherlands
Peru
Austria
South Sudan
United States
Kenya
Germany
Senegal
ElSalvador
Spain
Tanzania
Norway
Equatorial Guinea
Puerto Rico
(F) g_Gemella
LogRA
-1
-2
-3
Norway
ElSalvador
Austria
Netherlands
Puerto Rico
Spain
Tanzania
Ecuador
Germany
Peru
Senegal
Kenya
South Sudan
Mexico
Equatorial Guinea
United States
(G) g_Lactobacillus
LogRA
0
-1
-2
-3
Kenya
Senegal
Germany
Austria
South Sudan
Netherlands
Norway
Ecuador
Tanzania
Mexico
Peru
Puerto Rico
Spain
Equatorial Guinea
ElSalvador
United States
(H) g_Muribaculaceae
LogRA
-1
-2
-3
Kenya
Germany
Norway
Senegal
Peru
Ecuador
Mexico
Netherlands
Austria
Spain
United States
South Sudan
Equatorial Guinea
ElSalvador
Tanzania
Puerto Rico
(I) g_Prevotella
LogRA
-1
-2
-3
Mexico
Ecuador
Netherlands
Austria
Peru
United States
ElSalvador
Germany
Senegal
Spain
Kenya
South Sudan
Puerto Rico
Norway
Tanzania
Equatorial Guinea
(J) g_Pseudomonas
LogRA
0
-1
-2
-3
United States
ElSalvador
Peru
Tanzania
Kenya
Ecuador
Puerto Rico
Netherlands
Mexico
Austria
Spain
Senegal
Norway
Equatorial Guinea
South Sudan
Germany
(K) g_Staphylococcus
LogRA
0
-1
-2
-3
Norway
South Sudan
Netherlands
Spain
Senegal
Germany
Austria
Kenya
Ecuador
Peru
Puerto Rico
United States
ElSalvador
Mexico
Tanzania
Equatorial Guinea
(L) g_Streptococcus
LogRA
0
-1
-2
-3
ElSalvador
Peru
Austria
Netherlands
Norway
Senegal
Ecuador
Spain
Tanzania
Kenya
Germany
Puerto Rico
South Sudan
Mexico
United States
Equatorial Guinea
